# Endoscopic septoplasty versus conventional septoplasty for nasal septum deviation: a systematic review and meta-analysis of randomized clinical trials

**DOI:** 10.1097/MS9.0000000000000984

**Published:** 2023-06-20

**Authors:** Bayan O. Besharah, Hussain A. Alharbi, Omar A. Abu Suliman, Hazem K. Althobaiti, Ahmed M. Mogharbel, Sumaiya H. Muathen

**Affiliations:** a Department of Otolaryngology-Head and Neck Surgery, King Abdullah Medical Complex; b Department of Otolaryngology-Head and Neck Surgery, King Fahd Armed Forces Hospital, Jeddah; c Department of Nose, Sinus and Skull Base, King Abdullah Medical City, Makkah; d Department of Otolaryngology-Head and Neck Surgery, Al-Hada Armed Forces Hospital, Taif; e King Abdelaziz University, Rabigh Medical Collage, Saudi Arabia

**Keywords:** conventional septoplasty, deviated nasal septum, endoscopic septoplasty, randomized clinical trials, systematic review, meta-analysis

## Abstract

Although conventional septoplasty is widely used to treat nasal septum deviation, it increases morbidity due to poor visualization, poor illumination, the need for nasal packing, and difficulty in evaluating of the exact pathology. These drawbacks are also encountered in endoscopic septoplasty. Our study aimed to compare the treatment and complication outcomes of conventional and endoscopic septoplasty.

**Methods:**

The authors searched five electronic databases for relevant clinical trials. The records were screened for eligibility. Data were extracted from the included studies. Outcomes were pooled as risk ratios (RR) or mean differences with 95% CIs using RevMan ver.5.4.

**Results:**

Our study included 13 randomized clinical trials with 735 patients. Our analysis revealed that endoscopic septoplasty was significantly (*P*<0.05) superior to conventional septoplasty for postoperative nasal obstruction relief, intraoperative and postoperative hemorrhage, and mucosal adhesion and synechiae across both long-term and short-term follow-ups. The following pooled RR values were found in short-term follow-up periods: [RR=1.20, 95% CI:=(1.09,1.32)]; [RR=0.27, 95% CI=(0.14,0.54)]; and [RR=0.16, 95% CI=(0.08,0.32)], respectively. Regarding persistent septal deviation and septal tear, endoscopic septoplasty had the upper hand only in short-term follow-up periods [RR=0.30, 95% CI=(0.17,0.53)] and [RR=0.26, 95% CI=(0.15,0.46)], respectively.

**Conclusion:**

Our analysis revealed that endoscopic septoplasty was significantly superior to conventional septoplasty in postoperative nasal obstruction relief rate and reducing the risk of intraoperative and postoperative hemorrhage, mucosal adhesion and synechiae, persistent septal deviation, septal tear, and surgery duration.

## Introduction

Highlights

- Both conventional and endoscopic septoplasty have drawbacks.
- Our review evaluated the latest randomized clinical trial of conventional and endoscopic septoplasty.
- Endoscopic septoplasty resulted in overall better patient outcomes.
- However, most studies had moderate evidence; more well-designed research is needed in this field.

The prevalence of a straight nasal septum is low, as it is highly susceptible to trauma in intrauterine life^1^. Globally, only 15% of women and 7% of men have a straight nasal septum^2^. A deviated septum can be asymptomatic or induce functional and esthetic abnormalities. A deviated nasal septum can also contribute to infection due to poor ventilation of the paranasal sinuses. Consequently, a deviated septum should be corrected if it causes functional or cosmetic problems^3^.

Killian *et al.* and Freer *et al.* independently developed and refined the concept of submucosal excision early in the previous century. Conventional septoplasty, first described by Cottle *et al.* in 1947, is a traditional surgery in which only the deviated part of the septum is removed, leaving as much cartilage and bone as possible^4^. However, this procedure has increased morbidity owing to poor visualization, poor illumination, relative inaccessibility, the need for nasal packing, and difficulty in evaluating the exact pathology^2^.

The endoscopic repair of septal deformities was first introduced by Lanza and Zinriech in 1991^5^. Compared to traditional septoplasty, endoscopic septoplasty has many advantages, including improved visualization, accurate flap dissection with the removal of isolated deformities, a reduced risk of flap tears, and a smoother transition when combined with endoscopic sinus surgery^6,7^. However, despite these advantages, endoscopic septoplasty can be challenging because of frequent staining of the endoscope lens by blood from the incision site and difficulty in navigating the nasal passages^8^.

Evidence is required to assess the superiority of endoscopic septoplasty over conventional procedures. A previous meta-analysis by Hong *et al.* ^9^ included many low-quality nonindexed and observational studies in their pooled analysis, leading to potentially inconclusive results. In addition, there are newly published studies that compare both procedures. Therefore, in our study, we aimed to evaluate the treatment and complication outcomes of endoscopic septoplasty compared to those of conventional septoplasty.

## Methods

The study was designed according to the Cochrane Handbook for Systematic Review of Interventions and reported following the Preferred Reporting Items for Systematic Reviews and Meta-analyses (PRISMA), Supplemental Digital Content 1, http://links.lww.com/MS9/A160 and (AMSTAR), Supplemental Digital Content 2, http://links.lww.com/MS9/A161 guidelines^10–12^. According to AMSTAR 2, our systematic review and meta-analysis had a high quality. The protocol of our study was registered through PROSPERO (register number: CRD42022353987).

### Database search

We searched the Web of Science, PubMed, Scopus, Cochrane CENTRAL, and EMBASE from inception until August 2022. Additionally, all references listed in all eligible articles and any prior meta-analyses on the same topic were retrieved to identify any other missed relevant citations. The following search terms were used: (‘conventional’ OR ‘traditional’ OR ‘open’) AND (‘endoscopic’) AND (‘septoplasty’ OR ‘septal deviation’ OR ‘deviated nasal septum’ OR ‘nasal obstruction’).

### Eligibility criteria and screening

Two reviewers independently screened the retrieved references according to the eligibility criteria. The following criteria were applied to include the studies in our systematic review: trials whose patients suffered from any type of septal deviation; studies whose intervention was endoscopic septoplasty; studies in which comparator groups received conventional septoplasty; studies that assessed any of the following outcomes: postoperative nasal obstruction relief, postoperative nasal discharge relief, postoperative contact point headache relief, postoperative improvement in nasal obstruction and septoplasty effectiveness (NOSE) score, intraoperative and postoperative hemorrhage, mucosal adhesions and synechiae, persistent deviation, and septal tear; and randomized control trial (RCT) study design.

We excluded different studies for the following reasons: studies published in journals not indexed in Web of Science, PubMed, Scopus, Cochrane, or EMBASE; animal studies; nonrandomized trials and observational studies; studies that were not in English; abstract only; and study data that were not reliable for extraction and analysis.

### Data extraction

Data extraction was performed using an offline data extraction sheet after further checking to avoid any inclusion of data published in duplicate. The following data were extracted: study ID (first author and publication year), surgical technique, site, follow-up period, age, sex, intraoperative time, deviation type, NOSE score, inclusion criteria, conclusions, and main outcomes, which were as follows: Primary outcomes: postoperative nasal obstruction relief, intraoperative and postoperative hemorrhage, mucosal adhesions and synechiae, and persistent deviation. Secondary outcomes: postoperative nasal discharge relief, postoperative contact point headache relief, postoperative improvement in NOSE score, and septal tear.

### Risk of bias assessment

Two authors independently assessed the quality of the included trials using the Cochrane risk of a bias assessment tool for randomized controlled trials (RCTs)^13^. This tool comprises the following parameters: selection, performance, detection, attrition, reporting, and other possible sources of bias. The authors’ judgment was categorized as ‘high’, ‘low’, and ‘unclear’ risk of bias. Discrepancies were resolved through discussion or by a third assessor.

### Data synthesis

Regarding dichotomous outcomes, risk ratios (RR) were pooled with 95% CIs using the Mantel–Haenszel analysis method. Continuous outcomes were pooled as mean differences (MDs) between the two groups with 95% CIs using the inverse variance method. The fixed effects model was first applied if the effect estimate was pooled from homogenous studies; otherwise, the random effects model was applied. We investigated the statistical heterogeneity between studies using the *I* ^2^ statistics *χ* ^2^-test, with *P*>0.1 considered as heterogeneous and *I* ^2^ ≥ 50% suggestive of high heterogeneity. Additionally, wherever possible, we performed subgroup analyses of the outcomes reported after short-term (less than 6 months) or long-term (after one year) follow-ups to assess the results after each period. Publication bias assessment using funnel plots was performed for outcomes reported in 10 or more studies by visual inspection of the funnel plots. The Review Manager software version 5.4. was used for all statistical analyses.

## Results

### Literature search

**Figure 1:**
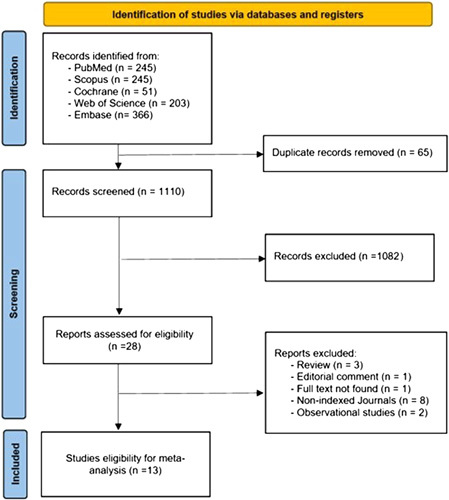
The PRISMA 2020 flow diagram illustrating the search process and study selection.

Our search method using four databases resulted in 1175 studies. After duplicate elimination, 1110 studies were eligible for screening. After title and abstract screening, 28 articles were found reliable for full-text screening. We rejected 15 of these; eventually, 13 articles met our criteria and were finally included in our analysis^8,13–24^. Figure 1 shows the PRISMA flow diagram for the study selection.

### Study characteristics

Our meta-analysis included 13 RCTs with a total of 735 patients; 359 patients in the conventional septoplasty group and 377 patients in the endoscopic septoplasty group^8,14–25^. The follow-up periods ranged from two months in Gulati *et al.* ^15^. to 24 months in Bothra Mathur^25^. Eight studies were conducted in India^8,14–16,18,22,23,25^, three in Egypt^20,21,24^, one in Israel^19^, and one in Canada^17^. Thirty-two patients suffered from C type septal deviation, 18 patients suffered from S type septal deviation, seven patients suffered from both types, 23 suffered from a right septal deviation, and 27 patients suffered from a left septal deviation; the remaining patients’ septal deviation types were not reported. Table 1 shows a detailed summary of the included studies and the baseline characteristics of their participants.

**Table 1:** Baseline characteristic of included studies.

References	Surgical technique, *n* (%)	Site	Age, (M±SD) years	Male, *n* (%)	Follow-up, months	Intraoperative time, (M±SD) min	Deviation types	NOSE score, (M±SD)	Inclusion criteria	Main outcomes	Conclusion
Doomra *et al*.^1^	Endoscopic septoplasty, 25 (50)	India	18–50 years	34 (68)	Up to 3	NR	1. C type 2. S type	NR	1. Patients aged 18–60 years 2. with symptomatic DNS such as nasal obstruction, hyposmia, nasal discharge, postnasal drip, and headache 3. with DNS complications 4. Informed consent given.	1. postoperative symptoms relieved 2. Postoperative complications.	‘ES is more effective in terms of relief of symptoms and improvement of nasal patency. It is best for isolated spur, posterior deviation, and revision surgery, but anterior caudal dislocation is best handled with CS. Both these techniques should be taken as an adjuvant to each other.’
	Conventional septoplasty, 25 (50)						1. C type 2. S type				
Gad *et al*.^2^	Endoscopic Septoplasty, 20 (50)	Egypt	28.6 ± 7.6	11 (50)	Up to 6	34.6±9.23	1.Septal spur 2. Broadly deviated septum 3. Multiseptal deformities.	NR	1. Male and female patients older than 16 years 2. With severe symptomatic DNS 3. complaining mainly of Nasal obstruction, headache, postnasal discharge and/or hyposmia.	1. postoperative symptoms relieved 2. Postoperative complications.	‘Endoscopic septoplasty is a valuable teaching tool, which is efficient in the management of different types of septal deformities.’
	Conventional Septoplasty, 20 (50).		28.4±6.8	8 (40)		50.9±9.03	1.Septal spur 2. Broadly deviated septum 3. Multiseptal deformities.				
Maini *et al*.^3^	Endoscopic Septoplasty, 50 (50)	India	NR	NR	4	NR	Posterior deviation	NR	1. Patients with nasal septal deviations 2. Major complaints were nasal obstruction Headache and discharge.	1. postoperative symptoms relief 2. Complications.	‘The clinical results of endoscopic septoplasty were found better as compared to conventional techniques with lesser complications and lesser periods of hospitalization. The use of endoscopic techniques offers lesser complications and lesser periods of hospitalization.’
	Conventional Septoplasty, 50 (50)										
Na’ara *et al*.^4^	Endoscopic Septoplasty, 30 (50).	Israel	31.5 ± 11.99	26 (86.6)	3	33.8+12	NR	NR	1. Patients older than 18 years 2. Underwent primary surgery for repairing DNS.	1. change in the SNOT-22 scores 2. Postoperative symptoms reliefs 3. Postoperative complications.	‘Endoscopic septoplasty and TNTS show similar results for treatment of nasal septum deviation’.
	Conventional Septoplasty, 30 (50)		24.1 ± 11.99	25 (83.3)		24.9+7.9					
Mandour *et al*.^5^	Endoscopic Septoplasty, 30 (50)	Egypt	Mostly (20–40)	38 (63.33)	3	NR	Anterior and posterior, NR.	NR	1. Sixty patients with the deviated nasal septum 2. With nasal obstruction and nasal discharge.	1. postoperative symptoms relief 2. Postoperative complications	‘Endoscopic septoplasty is a fast-developing concept and gaining popularity as it provides a direct targeted approach to the septal anatomic deformity, allowing a minimally invasive procedure with limited septal mucosal flap dissection and removal of small cartilaginous and/or bony deformity.’
	Conventional Septoplasty, 30 (50)										
Chandra *et al*.^6^	Endoscopic septoplasty, 25 (50)	India	28 ± 9.5	42 (84)	18	NR	1. DNS to the right side 2. DNS to the left side 3. C and S-shaped deviations.	NR	1. Age more than 14y 2. Patient with symptomatic DNS 3. With nasal obstruction, chronic rhinosinusitis.	1. postoperative symptoms relief 2. Postoperative complications	‘Endoscopic septoplasty has an obvious edge over the conventional approach due to better illumination which enables to identify the pathology accurately, excise the deviated part of the septum precisely and realignment of the cartilage for best results’.
	Conventional septoplasty, 25 (50).										
Sherif *et al*. ^7^	Endoscopic septoplasty, 15 (50)	Egypt	NR	NR	3	15 to 50	Anterior and posterior.	10.7 ± 4	1. An informed consent was obtained from the participants 2. Patients presented with nasal obstruction as main symptom 3. Underwent a full ENT examination.	1. postoperative symptoms relief 2. Postoperative complications 3. Postoperative NOSE score.	‘The endoscope allows precise resection of the pathological areas without the need of an extended dissection. It is associated with a significant reduction in the patient’s morbidity in the postoperative period. However, the endoscope has its own limitations which include loss of binocular vision and the need for frequent cleaning.’
	Conventional septoplasty, 15 (50)					1. Isolated septal spur,15 min 2. More than septal deformity,50 min.		10.5 ± 3.5			
Tukaram *et al*.^8^	Endoscopic septoplasty, 34 (65.38).	India	a. 11–20 years, 10 (29.41%) b. 21–30 years, 14 (41.17%) c. 31–40 years, 7 (20.58%) d. 41–50 years, 0 e. 51–60 years, 3 (8.82%).	39 (75)	3	NR	NR	NR	Patients with symptomatic DNS willing for surgery.	1. postoperative symptoms relief 2. Postoperative complications.	‘Endoscopic septoplasty allows accurate, conservative repair of obstructive nasal septum deviations, with fewer complications and better functional results compared to conventional septoplasty.’
	Conventional septoplasty,18(34.61)		a. 11–20y, 6(33.33%) b. 21–30y, 6(33.33%) c. 31–40y, 5(27.78%) d. 41–50y, 1(5.55%) e. 51–60y, 0								
Sathyaki *et al*.^9^	Endoscopic septoplasty, 25 (50).	India	33.44 ± 10.5	20 (80)	3	NR	1. Rt. DNS 2. Lt. DNS	NR	Patients with symptomatic DNS willing for surgery.	1. postoperative symptoms relief 2. Postoperative complications	‘Endoscopic septoplasty had better outcome with respect to complications. It is easier to correct posterior deviations and isolated spurs with endoscopic septoplasty. Complications are lesser with endoscopic septoplasty.’
	Conventional septoplasty, 25 (50)		29.88 ± 10	18 (72)			1. Rt. DNS 2. Lt. DNS				
Paradis *et al*.^10^	Endoscopic septoplasty, 32 (50.79).	Canada	48 ± 17.25	17 (53.12)	6	24 ± 7.8	S more than C shape.	14.7 ± 2.446	1. Patients diagnosed with a deviated septum 2. With Either C type or S type willing for surgery.	1. Postoperative NOSE score 2. Postoperative symptoms relief 3. Postoperative complications.	‘The endoscopic approach for septoplasty may be considered superior to the traditional approach for the correction of septal deviation.’
	Conventional septoplasty, 31 (49.2)		40 ± 10.75	22 (70.96)		52 ± 12.5	C more than S shape	15.2 ± 2.446			
Gulati *et al*.^11^	Endoscopic septoplasty, 25 (50).	India	72%, >25y	20 (80)	2	NR	NR	NR	1. Patients having symptomatic DNS 2. 18–40y after obtaining their consent.	1. postoperative symptoms relief 2. Postoperative complications.	‘The symptoms complained by the patients with pack in postoperative period and complications after surgery were significantly less in endoscopic septoplasty group.’
	Conventional septoplasty, 25 (50)		Mostly >25y	20 (80)							
Bothra *et al*.^12^	Endoscopic septoplasty, 40 (50).	India	8 to 42	48 (60)	12 to 24	NR	NR	NR	Patients with limited septal deviation and/or septal spur.	1. postoperative complications 2. Postoperative rhino metric findings.	‘No statistically significant difference was found between the conventional and endoscopic septoplasty groups, as assessed by subjective and objective evaluation.’
	Conventional septoplasty, 40 (50)										
Gupta et al.^13^	Endoscopic septoplasty, 25 (50).	India	NR	NR	15	NR	1. Cartilage, 7 (28) 2. Bone, 12 (48) 3. Both, 6 (24).	NR	1. 50 cases of DNS 2. Especially high and refractory to conservative medical treatment.	1. postoperative symptoms relief 2. Postoperative subjective improvement.	‘Endoscopic aided septoplasty was found to be safe, effective and conservative approach with better patient compliance, shorter recovery time and greater stability of remaining septum.’
	Conventional septoplasty, 25 (50)						1. Cartilage, 7 (28) 8 (32) 2. Bone, 12 (48) 12 (48) 3. Both, 6 (24) 5 (20).				

### Risk of bias assessment

Four of the included studies^14,17,19,25^ had a low risk of bias regarding randomization. However, although the remaining studies stated that they were randomized, they did not clarify if this involved random sequence generation or allocation. Twelve of our studies were open-label because of the nature of the procedures^8,14–16,18–25^. All the included studies had a low risk of bias regarding attrition and reporting. A summary of the risk of bias assessment is shown in (Supplementary Figure S1, Supplemental Digital Content 3, http://links.lww.com/MS9/A162).

### Efficacy outcomes

#### Postoperative nasal obstruction relief

**Figure 2:**
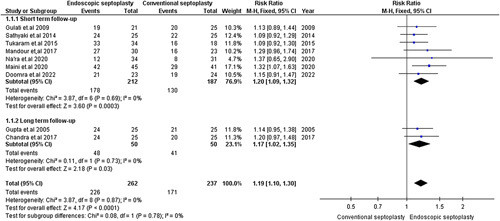
Postoperative nasal obstruction relief.

Relief of nasal obstruction was reported in nine studies with 499 patients^8,14–16,18–20,22,23^. The pooled RR of studies with short-term follow-up [RR=1.20, 95% CI=(1.09,1.32), *P*=0.0003] and long-term follow-up [RR=1.17, 95% CI=(1.02,1.35), *P*=0.03] significantly favored endoscopic septoplasty. The pooled studies were homogenous (short-term follow-up: χ^2^ *P*=0.69, *I* ^2^=0%; long-term follow-up: χ^2^ *P*=0.73, *I* ^2^=0%) (Fig. 2).

#### Postoperative nasal discharge relief

Eight studies, including 305 patients in total, reported postoperative nasal discharge relief^8,14–16,18,22–24^. The pooled RR did not favor either endoscopic or conventional septoplasty in the short- and long-term follow-up periods [RR=1.04, 95% CI=(0.94,1.14), *P*=0.43] and [RR=1.10, 95% CI=(0.94,1.29), *P*=0.25], respectively. The pooled studies with short-term and long-term follow-ups were homogenous (short-term: χ^2^ *P*=0.38, *I* ^2^=5%; long-term: χ^2^ *P*=0.52, *I* ^2^=0%) (Supplementary Figure S2, Supplemental Digital Content 3, http://links.lww.com/MS9/A162).

#### Postoperative contact point headache relief

**Figure 3:**
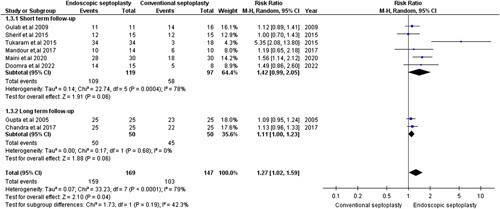
Postoperative Contact point headaches relief.

This outcome was reported in eight studies of 316 patients^8,14,15,18,20,22–24^. The pooled RR did not favor either endoscopic or conventional septoplasty in the short-term follow-up period [RR=1.42, 95% CI=(0.99,2.05), *P*=0.06], but the pooled studies were heterogeneous (χ^2^ *P*=0.0004, *I* ^2^=4%) (Fig. 3). However, we could resolve this heterogeneity by excluding Tukaram *et al.* ^22^. (χ^2^ *P*=0.28, *I* ^2^=21%); after heterogenicity was resolved, the pooled RR favored endoscopic septoplasty over conventional septoplasty [RR=1.23, 95% CI=(1.03,1.47), *P*=0.02] (Supplementary Figure S3, Supplemental Digital Content 3, http://links.lww.com/MS9/A162).

Among studies with long-term follow-up, there was no significant difference between the endoscopic and conventional groups [RR=1.11, 95% CI=(1.00,1.23), *P*=0.06]. The pooled studies were homogenous (χ^2^ *P*=0.68; *I* ^2^=0%) (Fig. 3).

#### Duration of surgery in minutes

The duration of surgery was reported in two studies with 103 patients^17,21^. The pooled MD favored endoscopic septoplasty over conventional septoplasty [MD=–22.21, 95% CI=(–33.67, –10.74), *P*=0.0001]. The pooled studies were not homogenous (χ^2^ *P*=0.003, *I* ^2^=89%); however, we could not resolve heterogeneity, as only two studies reported this outcome (Supplementary Figure S4, Supplemental Digital Content 3, http://links.lww.com/MS9/A162).

#### Postoperative improvement in NOSE score

Postoperative improvement in NOSE score was reported in two studies with 93 patients^17,24^. The pooled MD did not favor either endoscopic or conventional septoplasty [MD=–0.80, 95% CI=(–2.59,0.99), *P*=0.38]; the pooled studies were homogenous (χ^2^ *P*>0.00001, *I* ^2^=97%) (Supplementary Figure S5, Supplemental Digital Content 3, http://links.lww.com/MS9/A162).

### Complications

#### Persistent deviation

**Figure 4:**
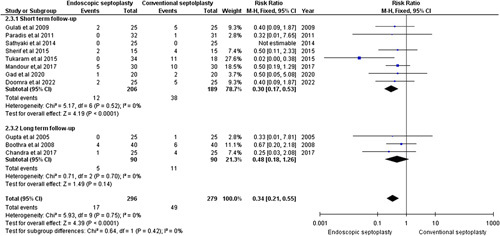
Postoperative persistent deviation.

Eleven studies, including 575 patients, reported persistent deviation^8,15–18,20–25^. Regarding the short-term follow-up period, endoscopic septoplasty was associated with a significantly lower incidence of persistent deviation than conventional septoplasty [RR=0.30, 95% CI=(0.17,0.53), *P*<0.0001]. The pooled studies were homogenous (χ^2^ *P*=0.52, *I* ^2^=0%). Regarding the long-term follow-up, our pooled analysis did not favor either endoscopic or conventional septoplasty [RR=0.48, 95% CI=(0.18,1.26), *P*=0.14]. The pooled studies were homogenous (χ^2^ *P*=0.70, I^2^=0%) (Fig. 4). Visual inspection of the asymmetry of the funnel plot revealed no significant publication bias (Supplementary Figure S6, Supplemental Digital Content 3, http://links.lww.com/MS9/A162).

#### Intraoperative and postoperative hemorrhage

**Figure 5:**
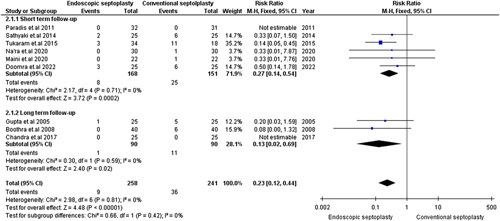
Intraoperative and postoperative heamorrhage.

Intraoperative and postoperative hemorrhage was reported in nine studies with 499 patients^8,14,16–19,22,23,25^. Endoscopic septoplasty was significantly associated with a lower incidence of intraoperative and postoperative hemorrhage than conventional septoplasty in the short-term [RR=0.27, 95% CI=(0.14,0.54), *P*=0.0002], as well as in the long-term [RR=0.13, 95% CI=(0.02,0.69), *P*=0.02]. The pooled studies were homogenous for both subgroups (short-term: χ^2^ *P*=0.71, *I* ^2^=0%; long-term: χ^2^ *P*=0.59, *I* ^2^=0%) (Fig. 5).

#### Mucosal adhesions and synechiae

Mucosal adhesions and synechiae were reported in ten studies involving 522 patients^8,15,16,18,20–25^. Endoscopic septoplasty was associated with a significantly lower incidence of mucosal adhesions and synechiae than conventional septoplasty in the short-term [RR=0.16, 95% CI=(0.08,0.32), *P*>0.00001] and the long-term [RR=0.21, 95% CI=(0.07,0.64), *P*=0.006]. The pooled studies were homogenous for both subgroups (short-term: χ^2^ *P*=0.82, I^2^=0%; long-term: χ^2^ *P*=0.98, *I* ^2^=0%) (Supplementary Figure S7, Supplemental Digital Content 3, http://links.lww.com/MS9/A162). Visual inspection of funnel plot asymmetry revealed no significant publication bias (Supplementary Figure S8, Supplemental Digital Content 3, http://links.lww.com/MS9/A162).

#### Septal tear

Six studies reported septal tears in 355 patients^8,14,16,17,21,22^. The pooled RR after short-term follow-up showed that endoscopic septoplasty significantly reduced the risk of septal tear compared with conventional septoplasty [RR=0.26, 95% CI=(0.15,0.46), *P*<0.00001]. The pooled studies were homogenous (χ^2^ *P*=0.25; *I* ^2^=25%) (Supplementary Figure S9, Supplemental Digital Content 3, http://links.lww.com/MS9/A162).

## Discussion

Our analysis based on 13 RCTs favored endoscopic septoplasty over conventional septoplasty in terms of postoperative nasal obstruction relief, duration of surgery, intraoperative and postoperative hemorrhage, mucosal adhesion, and synechiae. Regarding persistent septal deviation and septal tear, endoscopic septoplasty had the upper hand during the short-term follow-up period only. There was no statistically significant difference between traditional and endoscopic septoplasty in any of the other outcomes that were considered.

Our findings suggest that the endoscopic approach is preferable because of the endoscope’s enhanced illumination, which allowed for better visualization, limited flap elevation, minimized cartilage removal, allowed for good septal readjustment, and accurately symmetrically corrected septal abnormalities^2,26^.

The findings of previous literature investigating our studied outcomes were mostly consistent with our results^9^. Our findings indicated that endoscopic septoplasty was the preferred method for treating postoperative nasal obstruction, persistent septal deviation, septal tear, intraoperative, and postoperative bleeding, and mucosal adhesion and synechiae. We did not find a difference, but they did in terms of contact point headaches. Nevertheless, their pooled analysis did not match ours because they combined low-quality observational studies with RCTs published in nonindexed journals and did not separate the studies based on the length of time they followed the participants^9^.

Harley *et al.* ^27^ found that the surgical correction markedly improved in individuals with nasal blockage and headaches. Additionally, the correction of nasal symptoms, including nasal blockage and headache, was shown to be more successful in the endoscopic-assisted septoplasty group in a study by Nayak *et al.* ^28^. Removing the middle turbinate and other nasal structures with an endoscope at the same time is effective in some studies for relieving nasal discomfort^29^.

Our results are consistent with those of numerous other studies that have found synechiae formation to be more likely in patients who received conventional septoplasty as opposed to endoscopic septoplasty^23,30,31^. In one study by Talluri *et al.*, synechiae development was observed in 18.6% of the traditional septoplasty group and 4% of the endoscopic septoplasty group^29^.

Chandra *et al.* and Sherief *et al.* found that 4% of patients in the endoscopic group and 16% of patients in the conventional group had a persistently deviated nasal septum, which is similar to our findings^23,24^. Talluri *et al.* also revealed that 13.3% of conventional septoplasties and 6.6% of endoscopic septoplasties were found to involve partial repair^29^. However, some studies reported no difference between either of the two groups^16,17,21^; this could be due to their small sample size, which might have prevented them from detecting the difference.

Our study had the following strengths that promote it as strong evidence for the comparison of endoscopic and conventional septoplasty: our analysis was stratified according to follow-up duration. This was a very important point to address as some complications, such as synechiae formation, may take a long time to occur, and may lead to recurrent or incomplete deviation correction. Our evidence was based only on RCTs that were published in indexed journals to maximize the quality of the evidence. This was not the case in a previous meta-analysis on this topic^9^. Most of the pooled studies were homogenous. We only faced heterogeneity in two of our subgroups; we could resolve one of them (postoperative contact point headache relief) by sensitivity analysis. We identified publication bias when possible; this was not assessed in a previous meta-analysis^9^.

One of our limitations is the small sample size of most of our studied trials; moreover, no stratification was made regarding the preoperative status of patients or concomitant diseases with a deviated septum. Also, 12 of the 13 studies were open-label. Furthermore, mixed procedures like septoplasty, turbinoplasty, and FESS surgery were performed, which could have produced erroneous findings. Additionally, the results could be biased by variations in the surgeons’ skills and endoscopy experience. In addition, a small number of studies assessed the outcomes over long-term periods (<12 months), even though we had outcomes with no long-term assessment, or only one study assessed them. The quality of most of the included studies was moderate, with many unclear assessment domains, as they did not report enough data to permit judgment. The Cochrane Handbook for Systematic Reviews of Interventions guides interpreting the results of synthesis to communicate the conclusions of the review effectively. The handbook suggests that when interpreting the results of a systematic review, the quality of the included studies should be considered. For these reasons, the moderate quality of the included studies should be considered when interpreting the results.

Therefore, we recommend further well-designed studies with larger sample sizes, long-term assessment, and stratification of patients according to their preoperative status and concomitant diseases treated during surgery to obtain more powerful evidence.

## Conclusion

Our analysis revealed that endoscopic septoplasty was significantly superior to conventional septoplasty in increasing the postoperative nasal obstruction relief rate and reducing the risk of intraoperative and postoperative hemorrhage, mucosal adhesion and synechiae, persistent septal deviation, septal tear, and surgery duration.

## Ethical approval

NA.

## Consent

NA.

## Sources of funding

This research received no specific grant from any funding agency in the public, commercial, or not-for-profit sectors.

## Author contribution

The authors confirm contribution to the paper as follows: B.O.B.: study concept and design; H.A.A., H.K.A., A.M.M.: data collection; B.O.B., H.A.A.: writing the paper; O.A.A.S., S.H.M.: data interpretation and manuscript review; B.O.B.: principal investigator. All authors reviewed the results and approved the final version of the manuscript along with the editing and revision of comments.

## Conflicts of interest disclosure

The authors declare that there is no conflict of interest.

## Research registration unique identifying number (UIN)

This paper is registered through PROSPERO (register number: CRD42022353987.

## Guarantor

Besharah, Bayan Osama. MBBS,SBORL, JBORL, EBEORL-HNS. Otolaryngology-Head and Neck Surgeon. King Abdullah Medical Complex. Tel: 00966 564447097. E-mail: Bayan.o.b@gmail.com.

## Data availability statement

All data are available from the corresponding author upon reasonable request.

## Provenance and peer review

Not commissioned, externally peer reviewed.

## Supplementary Material
