# Supplementary material for: Endoscopic septoplasty versus conventional septoplasty for nasal septum deviation: a systematic review and meta-analysis of randomized clinical trials
Source: Ann Med Surg (Lond). 2023 Jun 20;85(8):4015–25. doi: 10.1097/MS9.0000000000000984 (PMC10406065; doi:10.1097/MS9.0000000000000984)
Supplement: Supplementary file 3 [file ms9-85-4015-s003.docx]

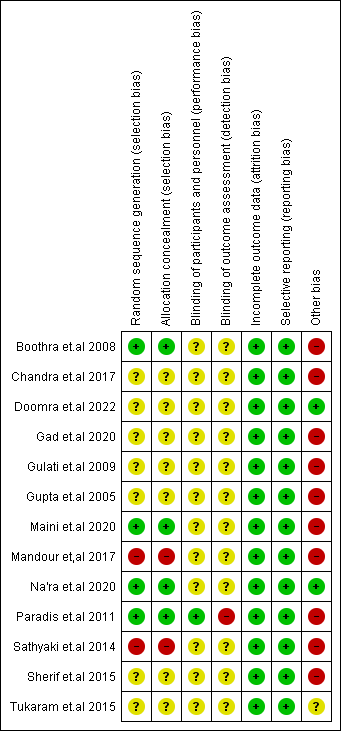


***Supplementary*** ***Figure S1:*** A summary of the risk of bias assessment.


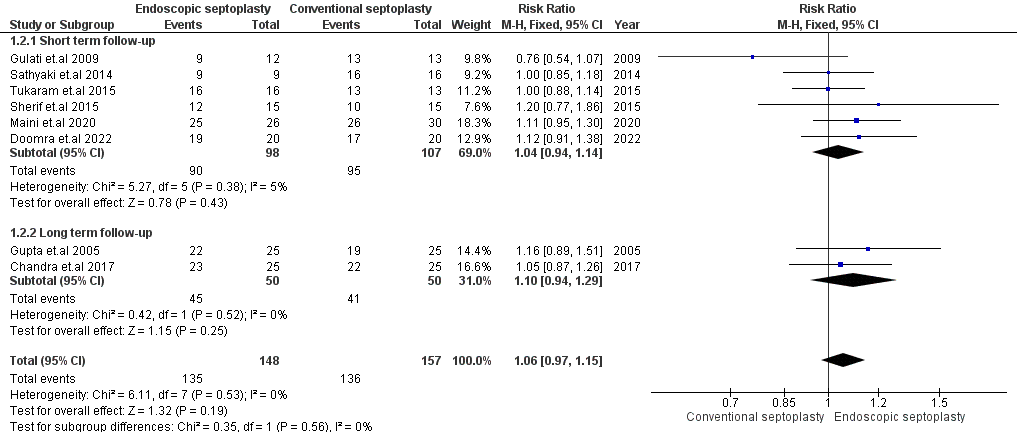


**Supplementary Figure S2:** Post-operative nasal discharge relief.


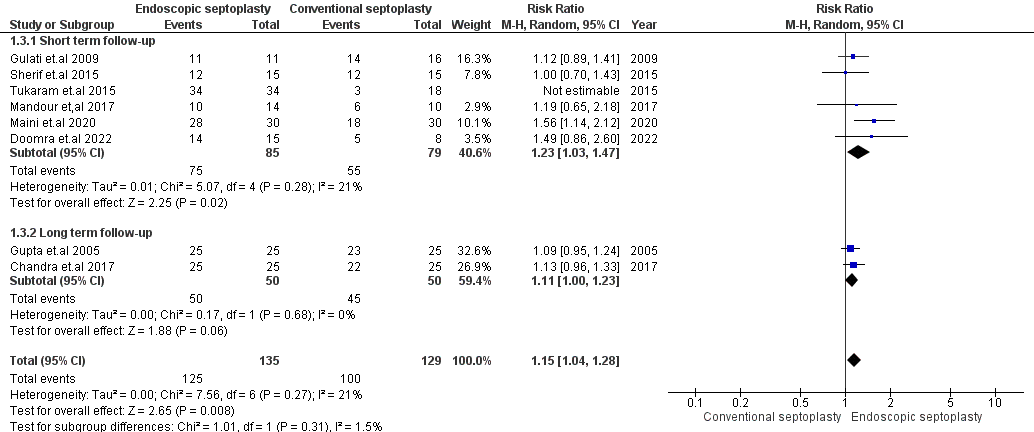


**Supplementary Figure S3:** Post-operative Contact pont headaches relief (After heterogenity).


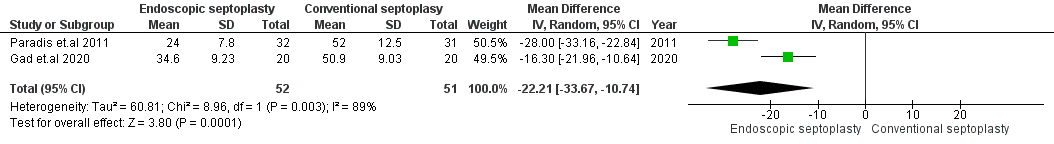


**Supplementary Figure S4:** Duration of surgery.


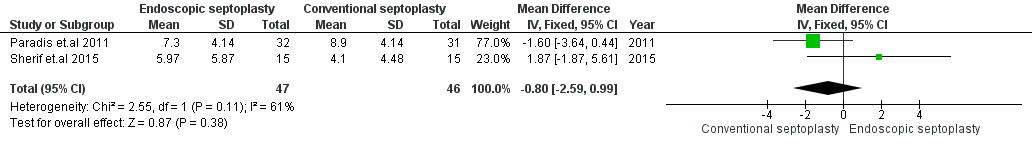


**Supplementary Figure S5:** NOSE score.


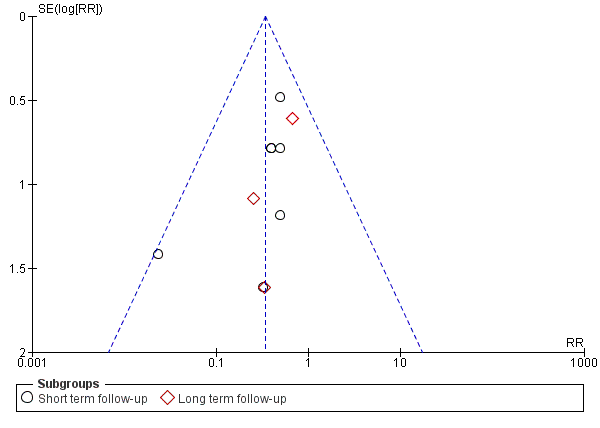


**Supplementary Figure S6:** Publication bias of Persistent deviation.


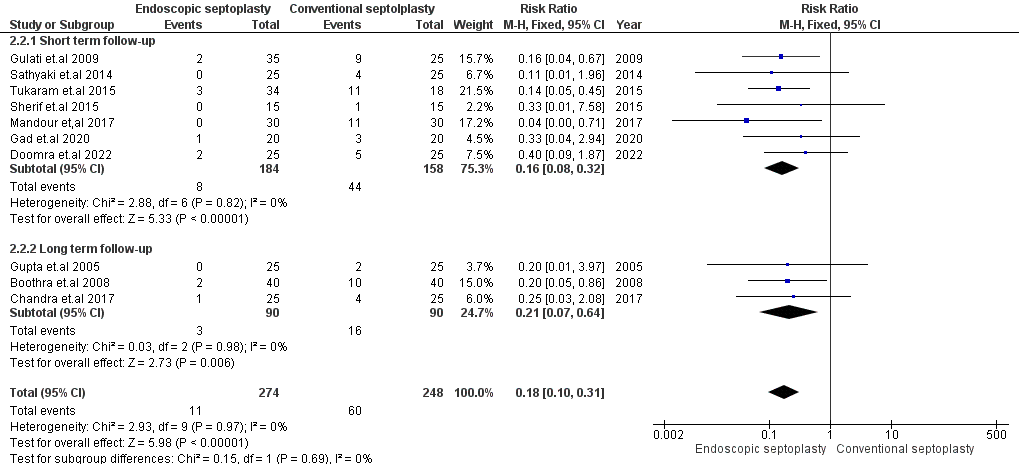


**Supplementary Figure S7:** Mucosal adhesions and synechiae.


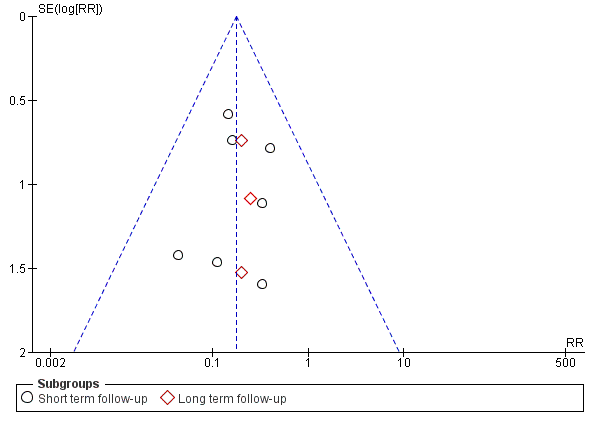


**Supplementary Figure S8:** Publication bias of Mucosal adhesions and synechiae.


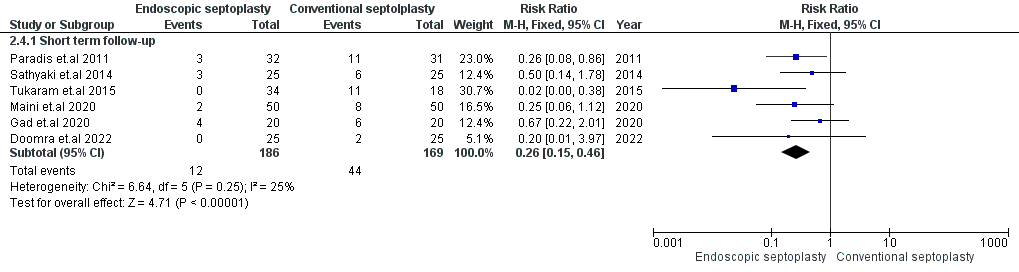


**Supplementary Figure S9:** Septal tear.
